# Feasibility of action observation effect on gait and mobility in idiopathic normal pressure hydrocephalus patients

**DOI:** 10.1590/1980-57642021dn15-010008

**Published:** 2021

**Authors:** Htet Htet Hnin, Sunee Bovonsunthonchai, Theerapol Witthiwej, Roongtiwa Vachalathiti, Rattapha Ariyaudomkit

**Affiliations:** 1 Faculty of Physical Therapy, Mahidol University – Nakhon Pathom, Thailand.; 2 Gait and Balance Group, Faculty of Physical Therapy, Mahidol University – Nakhon Pathom, Thailand.; 3 Division of Neurosurgery, Department of Surgery, Faculty of Medicine Siriraj Hospital, Mahidol University – Bangkok, Thailand.

**Keywords:** hydrocephalus, normal pressure, observation, movement, gait, walking, hidrocefalia de pressão normal, observação, movimento, marcha, deambulação

## Abstract

Action observation (AO) has been proved to be of benefit in several neurological conditions, but no study has previously been conducted in idiopathic normal pressure hydrocephalus (iNPH).

**Objective:**

This study aimed to investigate the feasibility of AO in iNPH patients.

**Methods:**

A single-group pretest-posttest design was conducted in twenty-seven iNPH patients. Gait and mobility parameters were assessed using the 2D gait measurement in the timed up and go (TUG) test for two trials before and after immediate AO training. The outcomes included step length and time, stride length and time, cadence, gait speed, sit-to-stand time, 3-m walking time, turning time and step, and TUG. In addition, early step length and time were measured. AO consisted of 7.5 min of watching gait videos demonstrated by a healthy older person. Parameters were measured twice for the baseline to determine reproducibility using the intraclass correlation coefficient (ICC_3,1_). Data between before and after immediately applying AO were compared using the paired t-test.

**Results:**

All outcomes showed moderate to excellent test-retest reliability (ICC_3,1=_0.51 0.99, p<0.05), except for the step time (ICC_3,1_=0.19, p=0.302), which showed poor reliability. There were significant improvements (p<0.05) in step time, early step time, gait speed, sit-to-stand time, and turning time after applying AO. Yet, the rest of the outcomes showed no significant change.

**Conclusions:**

A single session of AO is feasible to provide benefits for gait and mobility parameters. Therapists may modify this method in the training program to improve gait and mobility performances for iNPH patients.

## RESUMO.

A observação de ação (OA) teve benefícios comprovados em diversas condições neurológicas, mas nenhum estudo foi conduzido anteriormente em Hidrocefalia de Pressão Normal idiopática (HPNi).

**Objetivo:**

O presente estudo teve como objetivo investigar a viabilidade da OA em pacientes com HPNi.

**Métodos:**

Um projeto de pré-teste e pós-teste de grupo único foi realizado em 27 pacientes com HPNi. Parâmetros de marcha e mobilidade foram avaliados por meio de parâmetros 2D para a medida da marcha com o teste timed up and go (TUG) com duas tentativas antes e imediatamente depois do OA. Os resultados incluíram comprimento e tempo do passo, comprimento e tempo da passada, cadência, velocidade da marcha, tempo para sentar-e-levantar, tempo de caminhada de 3 metros, tempo de virada e passo, e tempo do teste (TUG). Além disso, o comprimento do passo inicial e o tempo da etapa inicial foram medidos. A OA consistia em assistir 7,5 minutos de vídeos de marcha demonstrados por um idoso saudável. Os parâmetros foram medidos duas vezes para a linha de base para determinar a reprodutibilidade usando o coeficiente de correlação intraclasse (CCI_3,1_). Os dados entre antes e depois da aplicação imediata de OA foram comparados com o teste *t* pareado.

**Resultados:**

Todos os resultados mostraram confiabilidade teste-reteste moderada a excelente (CCI_3,1=_0,51 0,99, p<0,05), exceto para o tempo do passo (CCI_3,1=_0,19, p=0,302), que apresentou confiabilidade pobre. Houve melhorias significativas (p<0,05) no tempo do passo, tempo do passo inicial, velocidade da marcha, tempo sentar-e-levantar e tempo de virar após a aplicação de OA. Os demais resultados não mostraram nenhuma mudança significativa.

**Conclusões:**

Uma única sessão de aplicação de OA é viável para proporcionar benefícios aos parâmetros de marcha e mobilidade. Os terapeutas podem modificar esse método no programa de treinamento para obter desempenho de marcha e mobilidade para pacientes com HPNi.

## INTRODUCTION

Idiopathic normal pressure hydrocephalus (iNPH), also known as Hakim-Adams syndrome, is a potentially reversible neurodegenerative disease that is increasing steadily nowadays.^1^ However, it could be possible that recovery from this disease is related to the disease’s duration, severity, early diagnosis, and treatment.^2^ iNPH is caused by cerebrospinal fluid (CSF) retention in the ventricle leading to its enlargement and expanding to the related brain tissue areas.^2^ ^,^ ^3^ The iNPH prevalence reported in Sweden during 1986 2000 was 0.2% for ages of 70 79 years and 5.9% for 80 years and older.^4^ From the population-based study, 3.7% of elderly over 65 years had a greater iNPH prevalence than the other age groups. Furthermore, the study reported four times higher prevalence in older people 80 years and older than the ones 65 79 years old.^5^ It was concluded that the prevalence of iNPH increases with age.^4^ ^,^ ^5^ However, the reported number is likely to be underestimated due to the patients not having received an accurate diagnosis.^4^ In a hospital-based study, by using clinical symptoms, neuroimaging, and released CSF pressure in the diagnosis, the estimation of prevalence was 21.9/100,000.^2^

The clinical presentation triad of iNPH is defined as; 1) progressive gait and balance disturbance, 2) urinary incontinence, and 3) cognitive impairment.^1^ ^,^ ^2^ ^,^ ^6^ ^–^ ^8^ In these symptoms, gait and balance disturbance and cognitive impairment were detected for 88% in the patients.^9^ Approximately 12 60% of the patients showed all of the three clinical symptoms.^9^ ^,^ ^10^ Abnormal gait pattern was characterized as a magnetic gait with difficulty to initiate the step and disequilibrium, which is usually known as a crucial feature of frontal gait disturbance.^1^ ^,^ ^9^ Abnormal characteristics of iNPH frequently consist of small steps, short stride, slowness, decreased step height, and *en bloc* gait and turning, and some individuals have a broad-based gait pattern, imbalance, and outward foot rotation.^9^ These disturbances are supposed to be the result of malfunction of the cortical and subcortical brain areas^9^ and low perfusion in the periventricular white matter and prefrontal regions.^11^ Also, the increase of intracranial pressure leading to the stretch and compression of the nerve fibers of the corticospinal tract that supplies the lower limb muscle.^11^

Cognitive deficits in iNPH patients are often associated with impaired short-term memory, speech difficulty, and loss of interest in surrounding people and environment.^1^ Cognitive and behavioral disturbances are caused by fronto-subcortical dysfunction involving executive dysfunction, inattention, slow mental processing, and apathy.^2^ ^,^ ^12^ ^,^ ^13^ Among these disturbances, apathy is the most common behavioral disturbance associated with gait disorders and may affect the improvement of functions and activities in iNPH patients after CSF release.^14^ For the memory and orientation functions, greater preservation was found in iNPH patients than in patients with Alzheimer’s disease.^12^ ^,^ ^15^

Currently, a standard treatment for iNPH is CSF drainage with different types of shunt surgery such as the ventriculoperitoneal (VP), ventriculoatrial (VA), and lumboperitoneal (LP) shunts.^6^ ^,^ ^16^ It is utilized in patients who respond to CSF drainage, intended to improve clinical symptoms while avoiding over-drainage complication.^6^ ^,^ ^16^ Among various clinical symptoms, gait responded to surgery the most^17^ and is often used as a prognostic factor for disease progression.^17^ Although the symptoms were dramatically improved after shunt surgery, the extent of gait abnormality was still the same.^18^ The effectiveness of shunt surgery may last longer, ranging between 3 5 years in 28 91% of iNPH patients.^2^ However, there are few reports about the rehabilitation benefit in iNPH patients after shunt surgery.^2^

Action observation (AO) has become a unique rehabilitation tool to date for both neurological and non-neurological disorders.^19^ ^–^ ^23^ AO is based on the mirror neuron system (MNS), used in the rehabilitation program to recover motor control and learning by recruiting the neural structures that can perceive and execute the actions.^19^ Mirror neurons can be responsible for the mechanism linking to observing the action and its understanding and imitation.^24^ These neurons are active throughout movement initiation to complete execution after observing the movement by reorganizing existing motor skills and cortical changes for the muscles involved in the observed action.^24^ Observing the other person’s dynamic action can use couple action-perception systems closely and influence motor performance planning of their own equivalently.^25^ It can activate the specific cerebral areas such as the premotor cortex and inferior parietal lobule, which are connected together to form the fronto-parietal circuits in organizing the actions.^26^

The actual or imagined locomotion tasks can activate the central locomotion control system, which includes the action observation network (AON) and other corresponding brain areas.^27^ According to a review of neuroimaging study, the premotor cortex, prefrontal cortex, and superior and inferior parietal lobules are the activated brain areas involved in AON. This was the higher level of activation occurring when the individuals were asked to observe the movements immediately after its observation.^28^ In addition, AON was also likely to be activated prominently during the observation of familiar movements compared with unfamiliar movements.^28^ Walking and other kinds of mobility function such as sit-to-stand and turn are dynamic familiar movements that we behave in our daily life and that can shape perception stimuli through the visual system.^29^

Observation of other person’s performance can activate representational areas where AON exists. This can be activated more when the observer performs the actual movement together during watching^28^ ^,^ ^30^ and the attention of observers’ on stimuli can have control over the currently perceived movement.^29^ AO has usually been used and demonstrated to be of benefit for improving motor function and learning in several conditions.^19^ ^–^ ^24^ ^,^ ^26^ ^,^ ^28^ ^,^ ^30^ ^–^ ^34^ It can be practiced by observing the action alone (action observation; AO) or observing combined with movement execution (action observation-execution; AOE). From a recent study by Zhu et al.^35^ that investigated the effect of AO and AOE on motor-cortical activation using magnetoencephalography in stroke patients. They concluded that AOE likely provides a good strategy to stimulate more brain activation at the primary motor cortex (M1), rather than AO alone as observed by a significant reduction of M1 beta oscillatory activity.

From a systematic review article,^32^ there are a quite number of studies of AO in patients with chronic and acute stroke, Parkinson’s disease (PD), cerebral palsy, and other orthopedic conditions. However, none of the studies investigated the effect of AO in iNPH patients. Therefore, the objective of this study was to investigate the feasibility of an acute effect of AO on gait and mobility parameters in iNPH patients after shunt surgery. We hypothesized that significant improvement of gait and mobility parameters would be found after applying AO.

## METHODS

This study was the first study designed as a single group, pre- and post-test design. Participants were informed about study details and signed an informed consent form prior to participating in the study. The study was approved by the university ethical review board (MU-CIRB COA No, 2019/179.0907) and the hospital ethical review board (SIRB COA No. 691/2019). In addition, this study has also been approved by the Thai Clinical Trials Registry, and the clinical registration number is TCTR20191104003.

### Participants

**Figure 1:**
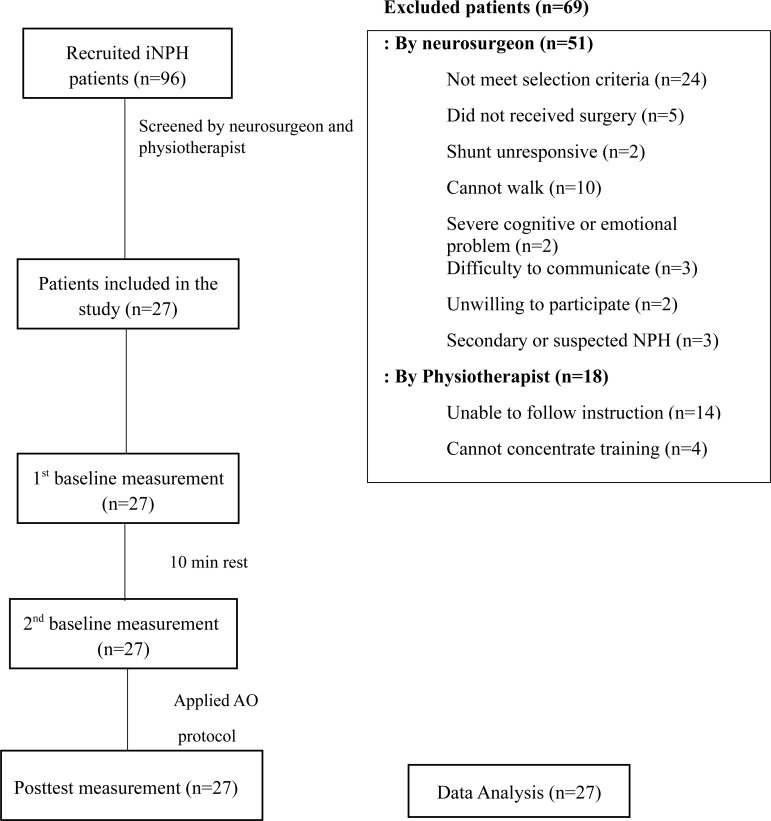
Flowchart of the study.

Ninety-six participants were recruited from the surgery unit, outpatient department, Siriraj Hospital, Bangkok, Thailand. For medical safety, participants were initially examined by the responsible neurosurgeon who provided the treatment before. They were then referred to screening following the study criteria with a well-trained physiotherapist. The inclusion criteria were age over 60 years, male or female, had received any kind of shunt surgery, able to follow the instructions, had no visual or auditory impairments after correction by glasses or hearing aid, and could walk with or without assistive device at least 10 m. Exclusion criteria were non-responsive to shunt surgery, unstable vital sign (blood pressure more than 160/90 mmHg and heart rate <59 beats/min or >90 beats/min), severe musculoskeletal problems such as severe osteoarthritis, deformities, and contractures in the lower limbs, severe pain that could affect gait performance and mobility, significant cognitive impairment, and unable to follow the instructions. Sixty-nine iNPH patients were excluded by the neurosurgeon (n=51) or physiotherapist (n=18). Hence, twenty-seven patients who met the selection criteria were left and took part in this study. They were checked for demographic characteristics, including age, weight, height, sex, and level of schooling. Afterwards, the clinical data were recorded, including the iNPH grading scale, Montreal Cognitive Assessment (MoCA), and time of shunt surgery. The flow chart diagram of the study is presented in Figure 1.

### Data collection placement and setting

**Figure 2:**
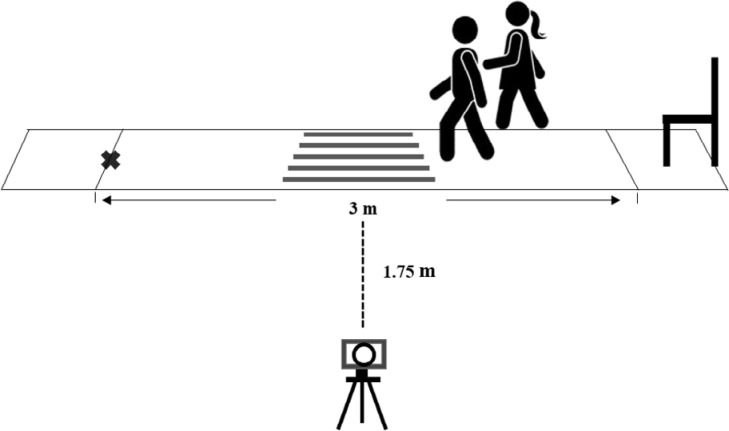
Data collection simulation.

Before collecting the data, the placement was prepared in a quiet room at the outpatient department of Surgery Unit, Siriraj Hospital, Bangkok, Thailand. Gait and mobility parameters were captured by the 2D measurement method modified from the previous study.^36^ Because some iNPH patients walked with a broad-based gait pattern, the walkway was created using five different referenced lines, each line was 50 cm in length. They were placed 7.2 cm apart, covered in the middle of the 3-m-long walkway. A video camera (Sony, HDR-CX210E, China) was placed at 1.75 m from the walkway perpendicularly. A standard chair was placed at the start point of the walkway. The data collection scenario is shown in Figure 2.

### Data collection protocol

Gait parameters were collected before and immediately after applied AO by a video camera. Prior to data collection, a research assistant who was a physiotherapist explained the details and demonstrated the timed up and go (TUG) test over the walkway. TUG is a widely used assessment tool to measure the effect of treatment on lower limb function and mobility^37^ ^–^ ^39^ and proposed to use in diagnosis criteria^40^ for iNPH. It demonstrated excellent reliability in elderly and chronic stroke.^41^ ^,^ ^42^ To ensure that the participants understood the gait capture protocol but avoided muscle fatigue, participants practiced their walking for one trial before collecting the real data. For the test-retest reliability analysis, gait data were collected for the first and second baselines with the two trials each, with a 10 min break or more between baseline capturing. Data between before and after immediately training were compared to investigate the training effect of AO. During testing, a physiotherapist walked behind the patient to prevent any hazardous situation such as a trip or fall and provide assistance in the required cases.

### Intervention

After finishing the second baseline measurement, participants received stretching and relaxation exercises together with the breathing exercise for 10–15 min for refreshment. Next, watching the video clips of AO which lasted 7.5 min, in which a healthy elderly person demonstrated the separated sequences of TUG which were captured from the front, back, left lateral, and right lateral views. The right lateral view captured a whole TUG movement. A video was edited by the combination of those demonstrated sequences with 30 s break between sequences. In addition, the model inside the videos performed the walking on the markers of the floor mat for each step.

The videos were prepared with different speeds, embedded with the sound from metronome at the frequency of 80, 85, 90, 95, and 100 beats/min (bpm). Each frequency can be attributed to different gait speeds: 0.65 m/s for 80 bpm, 0.75 m/s for 85 bpm, 0.85 m/s for 90 bpm, 0.9 m/s for 95 bpm, and 1 m/s for 100 bpm. The video was chosen at proper gait speed based on individual ability testing at the baseline, simply calculated over 3 m distance by using a stopwatch to record the time. It was selected with a minor challenging method by increasing speed by about 10–15% than the ability of the individual. The video was opened by a laptop computer (Lenovo, 15 inches), and then the participant was asked to sit on a chair at a place where they could clearly see the video. They were instructed to watch the video carefully and continuously and move their legs like marching in a sitting position, following the demonstrator in the video.

### Data tracking process

Spatiotemporal gait parameters and mobility parameters were collected at two time points (pre- and post-test). They included step length, step time, stride length, stride time, cadence, gait speed, early step length, early step time, sit-to-stand time, 3-m walking time, turning time, turning step, and TUG.

The data were analyzed by using the Kinovea Video Software, Windows Vista 10, version 0.8.15. The videos were opened inside the software and a 50-cm reference line nearest to the stepping foot was calibrated. According to the reference line, step length was tracked from a distance between the contact of opposite heel strike, the stride length from a distance between two successive points of the same heel contacts. These parameters were picked up from one or two gait cycles in the middle part of the walkway. While the early step length and early step time were extracted from the step first appearing on the screen.

Time concerning parameters were tracked by using a stopwatch item inside the software. The step time and stride time were tracked by the time taken of the step length and stride length. Sit-to-stand time was measured from sitting to standing up still, 3-m walking time was timed walking over 3-m walkway, turning time and step were captured while turning over 180 degrees, and TUG was the total time started from rise from a chair, walk 3 m, turn around, walk back to the chair, and sitting down. Gait speed was calculated over 3-m walkway divided by time spent, and cadence was calculated from 120 multiplied by gait speed and divided by stride length.

### Statistical analyses

Data were analyzed using the *Statistical Package for the Social Sciences* (SPSS) software (version 23) with the statistical significance level set at p<0.05. The Kolmogorov-Smirnov Goodness of Fit test was used and showed normal distribution. The descriptive statistic was used to describe demographic data and reported using mean and standard deviation. The intraclass correlation coefficient (ICC_3,1_) was used to determine the reproducibility of the testing protocol between the first and second baselines. The ICC values could be indicated as poor (0.00 0.50), moderate (0.50 0.75), good (0.75 0.90), and excellent (0.90 1.00) reliability.^43^ The data between before and immediately after AO training were compared using the paired t-test.

### Sample size calculation

The sample size was estimated from our pilot study (n=10) on the representative parameters for gait and sit-to-stand by the times to perform a step and sit-to-stand. A sample number was calculated using the G*Power software (version 3.1.9.2) with the t-tests function of comparing the difference between two dependent means (matched pairs). Determination of the alpha error probability 0.05 and power of 0.80 was set. The total sample sizes for step time, sit-to-stand time, and early step time were 7, 14, and 10, respectively. Hence, twenty-seven participants recruited in this study should cover and be sufficient to answer the research question.

## RESULTS

Demographics and clinical characteristics of the participants are presented in Table 1. Twenty-seven participants with mean age of 76.81±5.53, ranging from 65 to 85 years, including twenty-one males and six females, participated in the study. The mean weight and height were 63.34±12.89 kg and 162.59±7.21 cm. Most of the patients completed high school, and many patients had hypertension and diabetes mellitus as comorbidities. For the iNPH grading scale, all patients had gait disturbance (n=27) and most had cognitive impairment (n=24) and urinary problems (n=22). They were able to walk independently but were unstable (n=10) or walked with assistive devices (n=17). The mean MoCA score was 20.44±4.07, with scores ranging 12 to 28, and time post-shunt surgery was 1.84±2.35, ranging 0.06 to 11 years.

**Table 1:** Demographic data and clinical characteristics of the participants (n=27).

Parameters		Values
Age (years), mean±SD, range		76.81±5.53, 65.00–85.00
Weight (kg)		63.34±12.89, 44.50–92.60
Height (cm)		162.59±7.21, 148.00–176.00
Gender (male/female), n		21/6
Education, n		
	No study	1
	High school	14
	Bachelor’s degree	11
	Master’s degree	1
Comorbidities, n		
	Hypertension	19
	Diabetes mellitus	13
	Heart disease	4
	Parkinson’s disease	2
iNPH grading scale (scores), n		
Gait		
	0: Absent	0
	1: Unstable, but independent gait	10
	2: Walking with one cane	12
	3: Walking with two canes or walker frame	5
	4: Walking not possible	0
Cognition		
	0: Absent	3
	1: No apparent dementia, but apathetic	16
	2: Socially dependent, but independent at home	6
	3: Partially dependent at home	2
	4: Totally dependent	0
Urinary		
	0: Absent	5
	1: Absent, but with pollakisuria or urinary urgency	9
	2: Sometimes only at night	7
	3: Sometimes, even during the day	6
	4: Frequent	0
MoCA (scores), mean±SD, range		20.44±4.07, 12.00–28.00
Time since post-shunt surgery (years), mean±SD, range		1.84±2.35, 0.06–11.00

iNPH: idiopathic normal pressure hydrocephalus; MoCA: Montreal Cognitive Assessment.

### Test-retest reliability of the gait and mobility parameters between the first and second baseline measurements

Table 2 presents the reproducibility of gait and mobility parameters between the first and second baseline measurements. All parameters showed moderate to excellent test-retest reliability (ICC_3,1=_0.51 0.99, p<0.05), except for the step time, which showed no reliability (ICC_3,1=_0.19, p=0.302).

**Table 2:** Test-retest reliability of gait and mobility parameters between the first and second baseline measurements.

Parameters	First baseline (mean±SD)	Second baseline (mean±SD)	ICC_3,1_	95%CI	p-value*
Step length (cm)	35.07±8.75	35.67±8.11	0.98	0.955–0.991	**<0.001**
Step time (s)	0.61±0.09	0.62±0.06	0.19	0.786–0.629	0.302
Stride length (cm)	71.64±16.36	72.70±16.19	0.99	0.968–0.993	**<0.001**
Stride time (s)	1.26±0.16	1.22±0.11	0.67	0.286–0.852	**0.003**
Cadence (steps/min)	86.21±11.77	86.84±13.33	0.63	0.181–0.830	**0.007**
Gait speed (m/s)	0.51±0.11	0.53±0.11	0.95	0.890–0.977	**<0.001**
Early step length (cm)	31.24±10.18	32.17±10.17	0.86	0.696–0.937	**<0.001**
Early step time (s)	0.63±0.09	0.60±0.08	0.51	0.078–0.776	**0.038**
Sit-to-stand time (s)	2.21±1.17	1.94±0.84	0.92	0.829–0.965	**<0.001**
3 m walking time (s)	6.24±1.58	5.96±1.51	0.96	0.903–0.980	**<0.001**
Turning time (s)	3.19±1.03	3.08±1.07	0.85	0.678–0.933	**<0.001**
Turning step (steps)	5.31±2.04	5.07±2.31	0.94	0.875–0.974	**<0.001**
Timed up and go (s)	22.83±7.01	22.22±6.57	0.98	0.948–0.989	**<0.001**

* Statistical significance was tested by the ICC_3,1_ at p<0.05 (bold); SD: standard deviation; ICC: intraclass correlation coefficient; 95%CI: 95% confidence interval.

### Comparison of the gait and mobility parameters between before and immediately after action observation

Table 3 shows the comparison of gait and mobility parameters between before and immediately after AO. Significant differences were found in step time (p=0.002), gait speed (p=0.044), early step time (p=0.005), sit-to-stand time (p<0.001), and turning time (p=0.049), whereas the other parameters showed no change.

**Table 3:** Comparison of gait and mobility parameters between before and immediately after applied with action observation.

Parameters	Before (mean±SD)	Immediately after (mean±SD)	p-value*
Step length (cm)	35.37±8.35	35.22±7.81	0.769
Step time (s)	0.62±0.06	0.57±0.08	**0.002**
Stride length (cm)	72.17±16.15	73.09±16.14	0.206
Stride time (s)	1.24±0.12	1.21±0.13	0.238
Cadence (steps/min)	86.52±10.73	89.07±8.83	0.101
Gait speed (m/s)	0.52±0.11	0.54±0.12	**0.044**
Early step length (cm)	31.70±9.54	32.03±10.49	0.660
Early step time (s)	0.62±0.07	0.57±0.09	**0.005**
Sit-to-stand time (s)	2.08±0.98	1.74±0.79	**<0.001**
3 m walking time (s)	6.10±1.51	5.92±1.74	0.270
Turning time (s)	3.13±0.98	2.90±1.21	**0.049**
Turning step (steps)	5.19±2.12	4.94±2.37	0.095
Timed up and go (s)	22.53±6.72	22.07±8.11	0.366

* Significant difference tested by the paired t-test at p<0.05 (bold); SD: standard deviation.

## DISCUSSION

From our knowledge, this was the first study that investigated the effect of a single session of AO training on gait and mobility enhancement in iNPH patients post-shunt surgery. Except for the step time, the data showed test-retest reliability with a moderate degree in early step time, stride time, and cadence (ICC_3,1_=0.51–0.67), good in turning time and early step length (ICC_3,1_=0.85–0.86), and excellent in the other parameters (ICC_3,1_=0.92–0.99). This helps confirm to a certain extent that the findings may not come from testing repeatedly or the practice effect. As a result of the test-retest reliability results, we found that there was a variation for the step time, whereas the others showed relatively constant values in this patient population. This inconsistency may be linked to the common abnormal characters demonstrated in iNPH patients; freezing, shuffling or magnetic, and hesitant gait.^9^ ^,^ ^44^ ^,^ ^45^

After applying AO, significant improvements were found in step time (p=0.002), gait speed (p=0.044), early step time (p=0.005), sit-to-stand time (p<0.001), and turning time (p=0.049), and no change was found for the rest parameters. From a previous study, it was found that a single session of AO can induce an increase in spontaneous finger movement rate in PD.^22^ Furthermore, a systematic review about AO in various populations showed evidence that 5–6 min AO could be reasonable to sustain participants’ attention and enable training efficacy to improve motor function.^26^ In our study, we modified the AO protocol, which consisted in stretching and breathing exercises for refreshment and allowed the patients to execute the lower limb movement together with observing the video in a sitting position. These steps of the protocol were provided to prevent injury from walking tests and to maximize the effect of AO in brain stimulation but avoid fatigability in iNPH patients due to a frail body.

As the iNPH patients were older adults with kinds of locomotion deficit, most of them may not cope with long duration or heavy intensity of physical practice. A previous study demonstrated that AO alone could provide a beneficial effect on movement execution^46^ and increase walking performance in elderly people^47^ as well as in iNPH, which was shown in the present study. The results showed statistically significant improvements in time during walking (step and early step), sit-to-stand, turning, and gait speed, while other gait parameters such as step length, stride length, cadence, and timed capturing from a long distance as stride or TUG were not significant. This may be caused by the pathology of the ventricle enlargement and compress periventricular areas such as the internal capsule, corticospinal tract, and corpus callosum.^48^ Furthermore, as AO can only activate central mechanisms rather than peripheral, this can probably affect more the time parameters rather than the spatial.^47^

Relating to the characteristics of the participants, a wide range of post-shunt surgery duration (0.06 11 years) could affect the benefit of the intervention. The outcome of the efficacy of shunt surgery can be maintained for the short range between three and six months for 64–96% until one year for 41–95%, and a long term of three to five years for 28–91% of the patients. It showed that shunt surgery is predominantly effective up to five years at least. Taken together, the shunt surgery duration was not limited in this study; thus, the benefit of AO may also have been affected by this factor.^2^

On the other hand, we would expect that more sessions of AO in combination with strengthening exercise may be required to gain improvement noticeably on the temporospatial gait parameters for iNPH patients. There were different findings among the studies, depending on which parameters were selected and different training protocols.^20^ ^,^ ^31^ ^,^ ^33^ Step length, stride length, single support, cadence, and gait velocity were improved four weeks after AO in stroke patients.^20^ ^,^ ^33^ However, a study conducted in PD patients showed no significant improvement in stride length and walking speed after AO.^31^ Apart from the difference in training protocol, the controversial results among studies may result from the factors of different pathologies, brain changes, and clinical symptoms.

The study may have been limited by having only a single training session, small sample number, varied clinical symptoms relating to cognitive level and post-shunt surgery duration, and a single group without comparing to an age-matched control. Hence, an additional session of AO with a combined effect with another strengthening exercise program and a long-term assessment with randomized controlled trial should be conducted in future studies on iNPH patients.

In conclusion, this study shows that AO may be used in an iNPH population. A single session of AO slightly improved the temporal parameters of gait, sit-to-stand, and turn. Therapists may apply this strategy in the training program to enhance gait and mobility functions in iNPH patients.

## References

[1] Gavrilov GV, Gaydar BV, Svistov DV, Korovin AE, Samarcev IN, Churilov LP (2019). Idiopathic Normal Pressure Hydrocephalus (Hakim-Adams syndrome):
clinical symptoms, diagnosis and treatment. Psychiatr Danub.

[2] Mori E, Ishikawa M, Kato T, Kazui H, Miyake H, Miyajima M (2012). Guidelines for management of idiopathic normal pressure
hydrocephalus: second edition. Neurol Med Chir (Tokyo).

[3] Skalický P, Mládek A, Vlasák A, De Lacy P, Beneš V, Bradáč O (2020). Normal pressure hydrocephalus-an overview of pathophysiological
mechanisms and diagnostic procedures. Neurosurg Rev.

[4] Jaraj D, Rabiei K, Marlow T, Jensen C, Skoog I, Wikkelso C (2014). Prevalence of idiopathic normal-pressure
hydrocephalus. Neurology.

[5] Andersson J, Rosell M, Kockum K, Lilja-Lund O, Soderstrom L, Laurell K (2019). Prevalence of idiopathic normal pressure hydrocephalus: a
prospective, population-based study. PLoS One.

[6] Williams MA, Malm J (2016). Diagnosis and treatment of Idiopathic Normal Pressure
Hydrocephalus. Continuum (Minneap Minn).

[7] Damasceno BP (2009). Normal pressure hydrocephalus: diagnostic and predictive
evaluation. Dement Neuropsychol.

[8] Oliveira LM, Nitrini R, Roman GC (2019). Normal-pressure hydrocephalus: a critical review. Dement Neuropsychol.

[9] Souza RK, Rocha S, Martins RT, Kowacs PA, Ramina R (2018). Gait in normal pressure hydrocephalus: characteristics and
effects of the CSF tap test. Arq Neuro-Psiquiatr.

[10] Liew BS, Takagi K, Kato Y, Duvuru S, Thanapal S, Mangaleswaran B (2019). Current updates on Idiopathic Normal Pressure
Hydrocephalus. Asian J Neurosurg.

[11] Shprecher D, Schwalb J, Kurlan R (2008). Normal pressure hydrocephalus: diagnosis and
treatment. Curr Neurol Neurosci Rep.

[12] Picascia M, Minafra B, Zangaglia R, Gracardi L, Pozzi NG, Sinforiani E (2016). Spectrum of cognitive disorders in idiopathic normal pressure
hydrocephalus. Funct Neurol.

[13] Damasceno BP (2015). Neuroimaging in normal pressure hydrocephalus. Dement Neuropsychol.

[14] Allali G, Laidet M, Armand S, Saj A, Krack P, Assal F (2018). Apathy in idiopathic normal pressure hydrocephalus: a marker of
reversible gait disorders. Int J Geriatr Psychiatry.

[15] Picascia M, Zangaglia R, Bernini S, Minafra B, Sinforiani E, Pacchetti C (2015). A review of cognitive impairment and differential diagnosis in
idiopathic normal pressure hydrocephalus. Funct Neurol.

[16] Tudor KI, Nemir J, Pavliša G, Mrak G, Bilić E, Borovečki F (2020). Management of idiopathic normal pressure hydrocephalus (iNPH) - a
retrospective study. Br J Neurosurg.

[17] Ghosh S, Lippa C (2014). Diagnosis and prognosis in idiopathic Normal Pressure
Hydrocephalus. Am J Alzheimers Dis Other Demen.

[18] Katzen H, Ravdin LD, Assuras S, Heros R, Kaplitt M, Schwartz TH (2011). Postshunt cognitive and functional improvement in idiopathic
normal pressure hydrocephalus. Neurosurgery.

[19] Buccino G (2014). Action observation treatment: a novel tool in
neurorehabilitation. Philos Trans R Soc Lond B Biol Sci.

[20] Oh SJ, Lee JH, Kim DH (2019). The effects of functional action-observation training on gait
function in patients with post-stroke hemiparesis: a randomized controlled
trial. Technol Health Care.

[21] Park SD, Song HS, Kim JY (2014). The effect of action observation training on knee joint function
and gait ability in total knee replacement patients. J Exerc Rehabil.

[22] Pelosin E, Bove M, Ruggeri P, Avanzino L, Abbruzzese G (2013). Reduction of bradykinesia of finger movements by a single session
of action observation in Parkinson disease. Neurorehabil Neural Repair.

[23] Pazzaglia M, Galli G (2019). Action observation for neurorehabilitation in
apraxia. Front Neurol.

[24] Patel M (2017). Action observation in the modification of postural sway and gait:
theory and use in rehabilitation. Gait Posture.

[25] Cole GG, Welsh TN, Skarratt PA (2019). The role of transients in action observation. Atten Percept Psychophys.

[26] Sarasso E, Gemma M, Agosta F, Filippi M, Gatti R (2015). Action observation training to improve motor function recovery: a
systematic review. Arch Physiother.

[27] Griffa A, Van De Ville D, Herrmann FR, Allali G (2020). Neural circuits of idiopathic Normal Pressure Hydrocephalus: a
perspective review of brain connectivity and symptoms
meta-analysis. Neurosci Biobehav Rev.

[28] Mizuguchi N, Kanosue K (2017). Changes in brain activity during action observation and motor
imagery: their relationship with motor learning. Prog Brain Res.

[29] Scocchia L, Valsecchi M, Triesch J (2014). Top-down influences on ambiguous perception: the role of stable
and transient states of the observer. Front Hum Neurosci.

[30] Harris DJ, Vine SJ, Wilson MR, McGrath JS, LeBel ME, Buckingham G (2018). Action observation for sensorimotor learning in
surgery. Br J Surg.

[31] Jaywant A, Ellis TD, Roy S, Lin CC, Neargarder S, Cronin-Golomb A (2016). Randomized controlled trial of a home-based action observation
intervention to improve walking in Parkinson disease. Arch Phys Med Rehabil.

[32] Buchignani B, Beani E, Pomeroy V, Iacono O, Sicola E, Perazza S (2019). Action observation training for rehabilitation in brain injuries:
a systematic review and meta-analysis. BMC Neurol.

[33] Park HJ, Oh DW, Choi JD, Kim JM, Kim SY, Cha YJ (2017). Action observation training of community ambulation for improving
walking ability of patients with post-stroke hemiparesis: a randomized
controlled pilot trial. Clin Rehabil.

[34] Caligiore D, Mustile M, Spalletta G, Baldassarre G (2017). Action observation and motor imagery for rehabilitation in
Parkinson’s disease: a systematic review and an integrative
hypothesis. Neurosci Biobehav Rev.

[35] Zhu JD, Cheng CH, Tseng YJ, Chou CC, Chen CC, Hsieh YW (2019). Modulation of motor cortical activities by action observation and
execution in patients with stroke: an MEG study. Neural Plast.

[36] Aung N, Bovonsunthonchai S, Hiengkaew V, Tretriluxana J, Rojasavastera R, Pheung-Phrarattanatrai A (2020). Concurrent validity and intratester reliability of the
video-based system for measuring gait poststroke. Physiother Res Int.

[37] Bovonsunthonchai S, Witthiwej T, Ngamsombat C, Sathornsumetee S, Vachalathiti R, Muangpaisan W (2018). Effect of spinal tap test on the performance of sit-to-stand,
walking, and turning in patients with idiopathic normal pressure
hydrocephalus. Nagoya J Med Sci.

[38] Baltatanu D, Berteanu M (2019). Idiopathic Normal Pressure Hydrocephalus - what we
know. Maedica (Bucur).

[39] Wu EM, El Ahmadieh TY, Kafka B, Caruso JP, Neeley OJ, Plitt AR (2019). Clinical outcomes of normal pressure hydrocephalus in 116
patients: objective versus subjective assessment. J Neurosurg.

[40] Mendes GA, de Oliveira MF, Pinto FC (2017). The timed up and go test as a diagnostic criterion in normal
pressure hydrocephalus. World Neurosurg.

[41] Steffen TM, Hacker TA, Mollinger L (2002). Age- and gender-related test performance in community-dwelling
elderly people: Six-Minute Walk Test, Berg Balance Scale, Timed Up & Go
Test, and gait speeds. Phys Ther.

[42] Alghadir AH, Al-Eisa ES, Anwer S, Sarkar B (2018). Reliability, validity, and responsiveness of three scales for
measuring balance in patients with chronic stroke. BMC Neurol.

[43] Koo TK, Li MY (2016). A guideline of selecting and reporting intraclass correlation
coefficients for reliability research. J Chiropr Med.

[44] Ishikawa M, Yamada S, Yamamoto K (2019). Agreement study on gait assessment using a video-assisted rating
method in patients with idiopathic normal-pressure
hydrocephalus. PLoS One.

[45] Stolze H, Kuhtz-Buschbeck JP, Drucke H, Johnk K, Illert M, Deuschl G (2001). Comparative analysis of the gait disorder of normal pressure
hydrocephalus and Parkinson’s disease. J Neurol Neurosurg Psychiatry.

[46] Wulf G, Shea C, Lewthwaite R (2010). Motor skill learning and performance: a review of influential
factors. Med Educ.

[47] Tia B, Mourey F, Ballay Y, Sirandre C, Pozzo T, Paizis C (2010). Improvement of motor performance by observational training in
elderly people. Neurosci Lett.

[48] Siasios I, Kapsalaki EZ, Fountas KN, Fotiadou A, Dorsch A, Vakharia K (2016). The role of diffusion tensor imaging and fractional anisotropy in
the evaluation of patients with idiopathic normal pressure hydrocephalus: a
literature review. Neurosurg Focus.

